# Plasma exosomes induced by remote ischaemic preconditioning attenuate myocardial ischaemia/reperfusion injury by transferring miR-24

**DOI:** 10.1038/s41419-018-0274-x

**Published:** 2018-02-23

**Authors:** Wen Minghua, Gong Zhijian, Huang Chahua, Liang Qiang, Xu Minxuan, Wang luqiao, Zhang Weifang, Lu Peng, Zhan Biming, Yu Lingling, Wang Zhenzhen, Xu Jianqing, Bao Huihui, Wang Xiaozhong, Cheng Xiaoshu

**Affiliations:** 1 Department of Cardiovascular Medicine, The Second Affiliated Hospital of Nanchang University, Nanchang, China; 2 Jiangxi Province Key Laboratory of Molecular Medicine, Nanchang, China; 3 Department of Laboratory Medicine, The Second Affiliated Hospital of Nanchang University, Nanchang, China

## Abstract

Remote ischaemic preconditioning (RIPC) is well known to protect the myocardium against ischaemia/reperfusion injury (IRI). Exosomes are small extracellular vesicles that have become the key mediators of intercellular communication. Various studies have confirmed that circulating exosomes mediate RIPC. However, the underlying mechanisms for RIPC-induced exosome-mediated cardioprotection remain elusive. In our study, we found that the expression level of miR-24 was higher in exosomes derived from the plasma of rats subjected to RIPC than in exosomes derived from the plasma of control rats in vivo. The rat plasma exosomes could be taken up by H9c2 cells. In addition, miR-24 was present in RIPC-induced exosomes and played a role in reducing oxidative stress-mediated injury and decreasing apoptosis by downregulating Bim expression in H_2_O_2_-treated H9c2 cells in vitro. In vivo, miR-24 in RIPC-induced exosomes reduced cardiomyocyte apoptosis, attenuated the infarct size and improved heart function. Furthermore, the apoptosis-reducing effect of miR-24 was counteracted by miR-24 antagomirs or inhibitors both in vitro and in vivo. Therefore, we provided evidence that RIPC-induced exosomes could reduce apoptosis by transferring miR-24 in a paracrine manner and that miR-24 in the exosomes plays a central role in mediating the protective effects of RIPC.

## Introduction

Ischaemic heart disease and the resulting heart failure remain the leading causes of death and disability in worldwide. So novel therapies are required to protect the heart against the detrimental effects of acute ischaemia/reperfusion injury (IRI)^1^. It is well known that remote ischaemic preconditioning (RIPC) protects the myocardium against IRI, but the underlying mechanism remains elusive. Recently, it has been shown that extracellular vesicles (EVs) released from the heart after IPC are required for cardioprotection by RIPC^2^ and that endogenous plasma EVs can also protect the heart from IRI^3^. However, understanding the nature of the actual effector factors harboured in these vesicles needs further molecular and in vivo experimentation.

Exosomes are EVs, which are smaller than 150 nm in diameter. Exosomes are enriched in components, including RNA, microRNAs (miRNAs), proteins and lipids. EVs can be targeted to the recipient cells via their surface molecules. Once attached to the target cell, EVs can induce signalling via receptor–ligand interactions, and be internalised by phagocytosis and/or endocytosis or even fuse with the membrane of the target cell to deliver cargo into the cytoplasm, thus modifying the target cell’s physiological state^4^. Various cells can release miRNAs via exosomes, which can protect miRNAs from degradation and guarantee their stability and circulation through the bloodstream^5,6^.

miRNAs are highly conserved endogenous, small, noncoding RNA molecules, which have emerged as main posttranscriptional regulators of gene expression^7,8^. miRNAs negatively impact the expression of their target mRNAs through imperfect base-pairing and are well known to play a significant role in cellular proliferation, differentiation, apoptosis and stress responses in the cardiovascular system^9^.

The interactions of released exosomes with or uptake by the recipient cells are common forms of intercellular communication. However, limited information is available regarding the biological signals that are transported by exosomes. Among the various potential mechanisms, one interesting hypothesis is that the exosomes carry well-defined considerable of miRNAs, which relay specific biological information between cells. For example, recent studies have shown that stem cells play a cardioprotective effect by releasing miRNAs that are carried by exosomes to recipient cells^10,11^. Exosomes, acting as carrier EVs, play an important role in cellular communication through the exchange of proteins or miRNAs between cells^12^. However, it is essential to explore the potential biological functions of miRNA contents in exosomes released following RIPC^13^. Since the subject of our study was IRI, we selected nine miRNAs that have been reported to be involved either in oxidative stress (such as miR-150 and miR-21)^14,15^ or in cardiomyocyte apoptosis (such as miR-195, miR-132, miR-140, miR-144, miR-24, miR-214 and miR-34a)^16–21^ in our investigation and, using quantitative PCR (qPCR), explored whether RIPC could modify the expression level of these nine miRNAs in plasma exosomes. We observed that the expression of miR-24 was significantly higher in exosomes from the plasma of rats subjected to RIPC that in those from rats subjected to sham operation in vivo. Previous studies have demonstrated that miR-24 plays a cardioprotective role in myocardial infarction by downregulating the expression of the pro-apoptotic protein Bim.

Based on these observations, we proposed the hypothesis that RIPC-EXO transport miR-24 to cardiomyocytes to attenuate myocardial IRI by decreasing myocardial apoptosis via the downregulation of Bim expression.

## Results

### Collection and identification of exosomes induced by RIPC

**Fig. 1:**
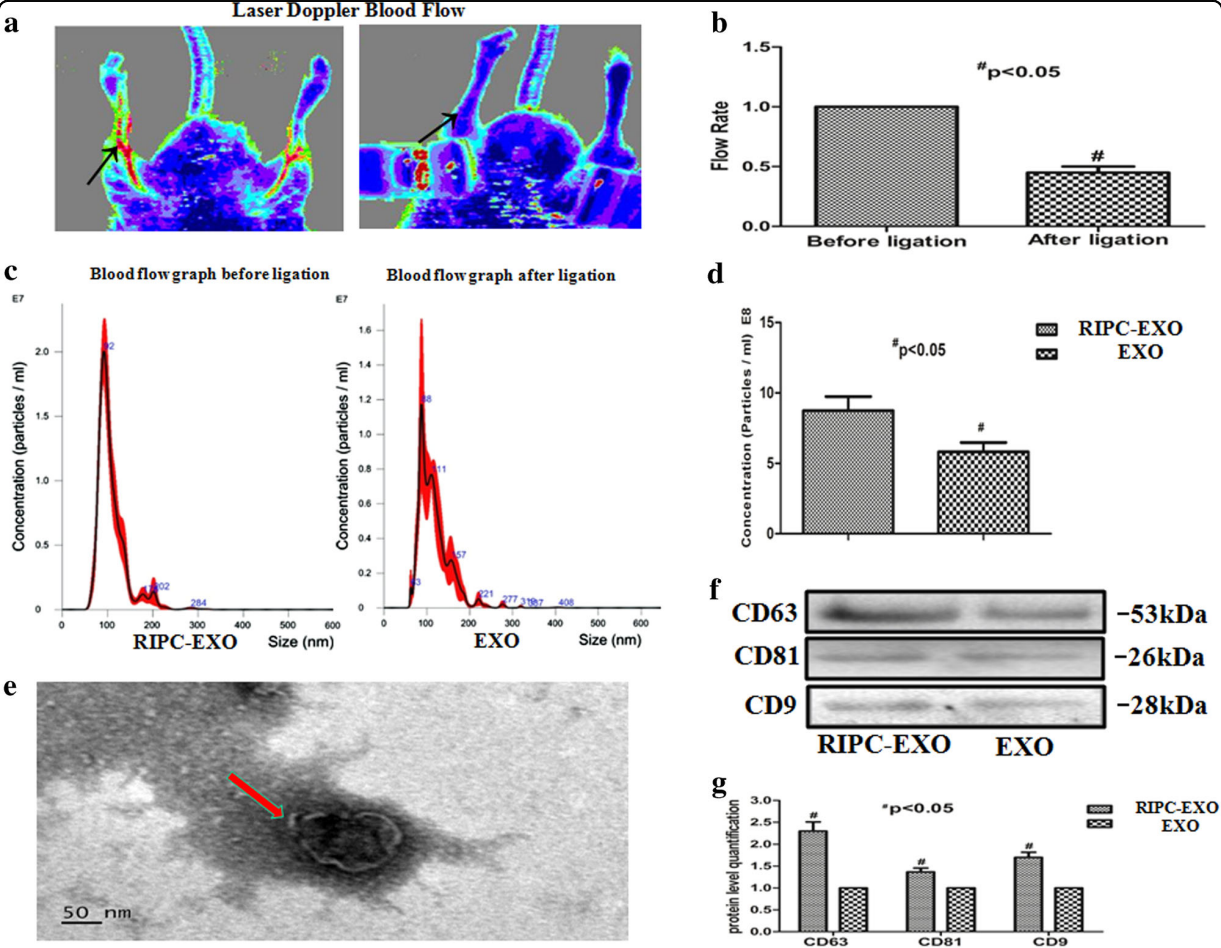
Establishment of the rat RIPC model and characterisation of plasma exosomes. **a** Transient ischaemia was bilaterally generated in rat hindlimbs using tourniquets. A reduction in blood flow during RIPC was confirmed by laser Doppler blood flow measurements. High perfusion is displayed as red, while low or negative perfusion is displayed as dark blue. **b** Computer-assisted quantitative analysis demonstrated a significant decrease in the flow rate after ligation (^#^*P* < 0.05 vs before ligation, *n* = 4). **c** NTA demonstrates the size distribution of EXO and RIPC-EXO derived from the same amount of plasma. **d** The concentration of the particles of RIPC-EXO was greater than that of the EXO (^#^*P* < 0.05, *n* = 4). **e** Electron micrographs of purified rat exosomes. Bar: 50 nm. **f** The presence of exosomal marker proteins, including CD63, CD81 and CD9 were confirmed by western blots in the rat samples. **g** Levels of the exosomal marker proteins CD63, CD81 and CD9 were increased after RIPC. Samples were derived from the same amount of plasma exosomes (^#^*P* < 0.05 vs EXO, *n* = 3)

We first used laser Doppler blood flow measurements to confirm transient hindlimb ischaemia induced by RIPC in rats (Fig. 1a, b). During the RIPC treatment (RIPC protocol was 4 × 5 min). The hindlimb blood flow was diminished, which was recovered after the tourniquet was released as previously reported^22^. The plasma samples were taken from the same rats before and 5 min after RIPC. The exosomes induced by RIPC were obtained from the plasma using Exoquick followed by fibrin clear to prevent its co-precipitation, and their morphology and phenotypes were determined based on exosome characterisation as previously described^23^. First, the size range and concentration of the particles were measured by using nanoparticle tracking analysis (NTA). The diameters of the almost all particles were between 50 and 200 nm, with an average value of 108 nm (Fig. 1c). Furthermore, the concentration of the particles of RIPC-EXO was significantly greater than that of the EXO from the same amount of plasma (8.75 ± 1.02 × 10^11^ vs. 5.81 ± 0.64 × 10^11^ particles/ml), suggesting that RIPC could increase the secretion of exosomes (Fig. 1d). The morphology of the RIPC-induced particles were directly examined via transmission electron microscopy (TEM). The results showed the particles were cup-shaped membrane-bound vesicles with a diameter of ~100 nm (Fig. 1e). Additionally, the expression of exosomal markers, including CD63, CD9 and CD81 was detected in RIPC-EXO by western blotting, and the levels of these markers were higher in RIPC-EXO than in EXO (Fig. 1f, g).

### Exosome labelling and uptake by H9c2 cells

**Fig. 2:**
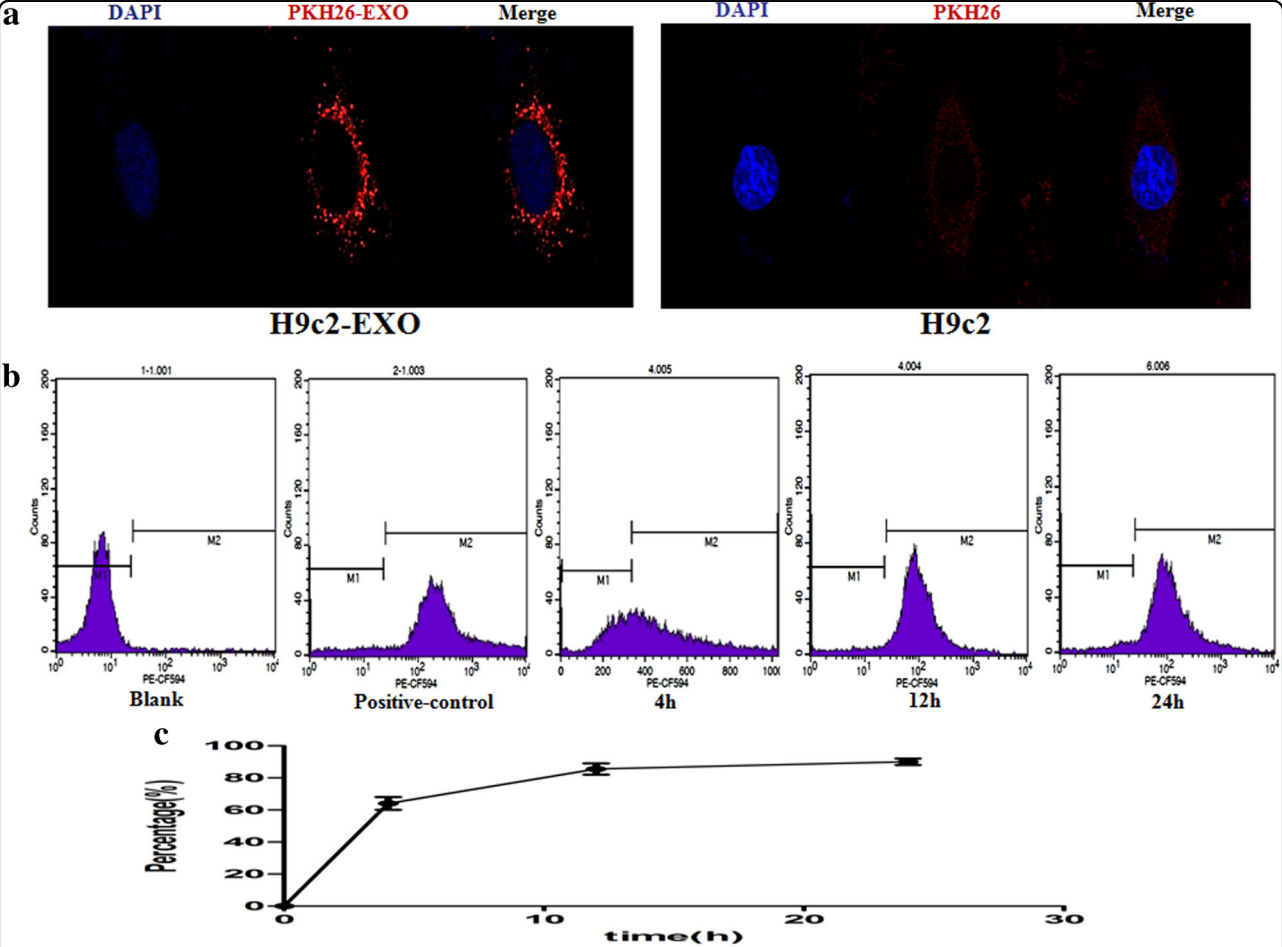
Exosome labelling and uptake by cells in vitro. **a** Representative confocal microscopy of H9c2 cells that was exposed to PKH26-labelled plasma exosomes from RIPC-treated rats. H9c2 cells incubated with only PKH26 served as negative controls. Nuclei were stained with DAPI (blue). Red: PKH26. **b**, **c** Flow cytometric analysis of the uptake of exosomes by H9c2 cells at various time points

To conform whether RIPC-EXO could be taken up by H9c2 cells, RIPC-EXO were labelled with the PKH26 dye, which has a strong red fluorescence. Indeed, H9c2 cells incubated with PKH26-labelled exosomes exhibited red fluorescence in the cytoplasm (Fig. 2a), indicating the uptake of a significant amount of exosomes. In contrast, the negative control (NC) group only exhibited a weak red fluorescence signal. The typical uptake efficiency of exosomes was ~89% as determined by fluorescence-activated cell sorting (Fig. 2b, c).

### Potential involvement of miR-24 in RIPC-EXO in protecting cardiomyocytes against apoptosis

**Fig. 3:**
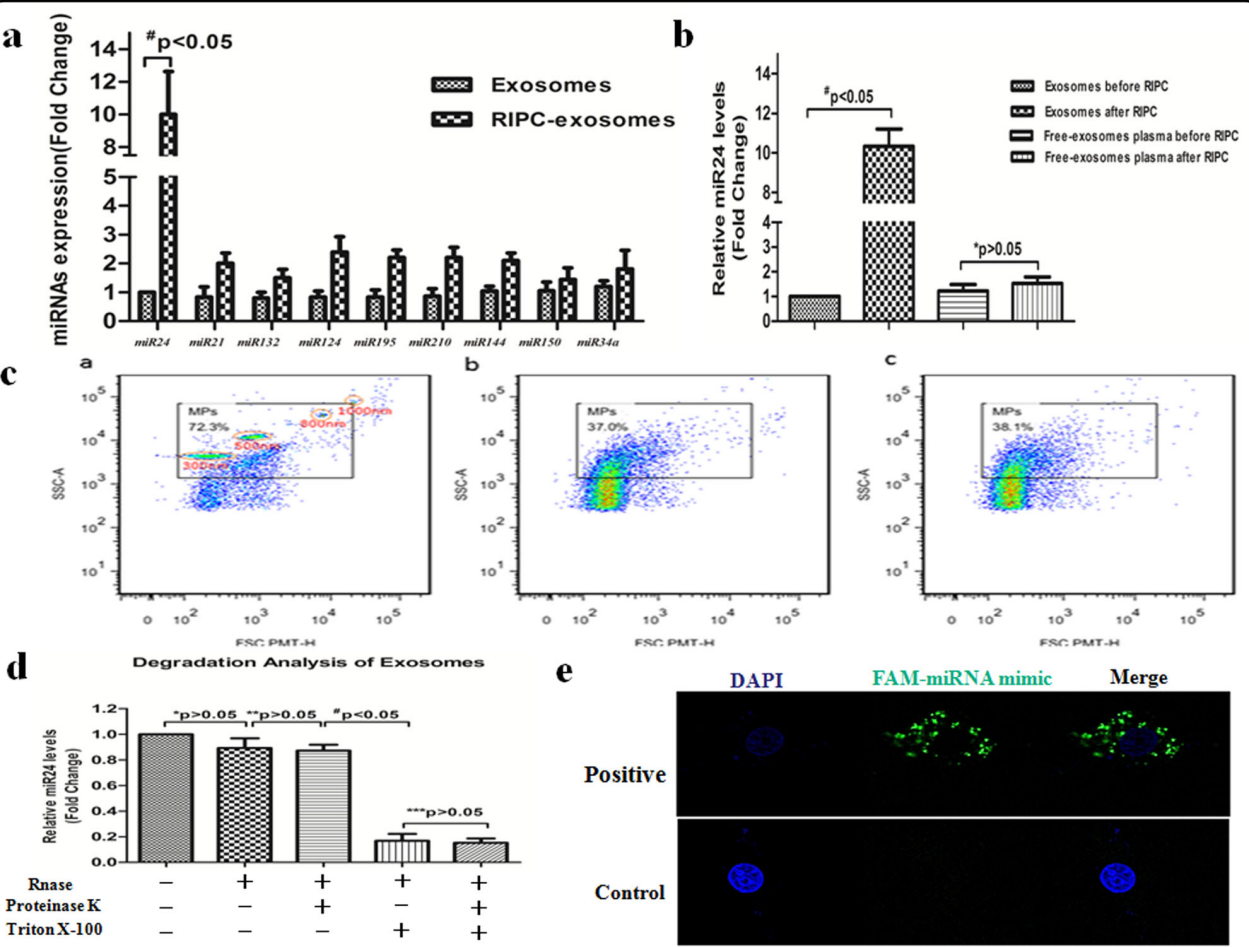
RIPC-induced expression of miR-24 in exosomes. **a** qPCR analysis of principal miRNAs that have been reported in exosomes from sham-operated and RIPC-treated rats. Nine miRNAs (miR-21, miR-24, miR-214, miR-132, miR-195, miR-210, miR-144, miR-150 and miR-34a) were found in exosomes obtained from rats subjected to RIPC, but only miR-24 was significantly upregulated (^#^*P* < 0.05, *n* = 4). **b** Levels of miR-24 in exosomes and exosome-free plasma before and after RIPC were determined by RT-PCR (*P > 0.05, ^#^*P* < 0.05, *n* = 4). **c** Flow cytometry was used to detect microcapsules in RIPC-treated rat plasma. a: Microcapusles are circled; b: before RIPC; c: after RIPC. Number of microcapsules did not significantly change before or after RIPC. **d** Exosomes were isolated from RIPC-treated rat plasma and incubated with the indicated reagents, after which RNA was isolated, and the expression levels of miR-24 were determined using qPCR (*P > 0.05, ^#^*P* < 0.05, *n* = 3). **e** Representative confocal microscopy images of H9c2 cells incubated with exosomes from H9c2 cells that had been transfected with FAM-labelled miR-24 mimics (unlabelled miRNAs were used as controls)

We further determined the expression of nine miRNAs (miR-24, miR-21, miR-214, miR-132, miR-195, miR-210, miR-144, miR-150 and miR-34a) in both RIPC-EXO and EXO (Fig. 3a). Among these miRNAs, miR-24 level was significantly higher in RIPC-EXO than in EXO purified from sham-operated rat plasma (Fig. 3b).

To confirm that miR-24 is predominantly present in exosomes rather than in other forms, we first measured the number of microcapsules in plasma by flow cytometry. However, the number of microcapsules did not change significantly before and after RIPC (Fig. 3c). Next, exosomes purified from plasma from rats subjected to RIPC were treated with proteinase K, RNase or Triton X-100 at 37 °C for 45 min. Indeed, miR-24 detected in plasma was mainly harboured within exosomes (Fig. 3d).

To demonstrate that exosomes were able to transport miR-24 to recipient cells, we fluorescently labelled miR-24 mimics and transfected them into cells, collected the exosomes secreted by the cells, and then incubated H9c2 cells with these exosomes. We found that H9c2 cells harboured fluorescently labelled miR-24 mimics (Fig. 3e).

As a common reactive oxygen species, H_2_O_2_ is widely used to mimic oxidative stress inducing cell death occurring during myocardial IRI in vitro experiments^24,25^. We thus examined the effect of H_2_O_2_ on the death of H9c2 cells and observed that treatment with 100 μmol/l H_2_O_2_ trigged apoptosis, whereas treatment with 600 μmol/l H_2_O_2_ preferentially caused necrosis (Supplemental Fig. IA). A time-dependent increase in the fraction of apoptotic H9c2 cells was observed following treatment with 100 μM H_2_O_2_ (Supplemental Fig. IB). Additionally, treatment with 100 μM H_2_O_2_ for 6 h significantly increased apoptosis in H9c2 cells as analysed by flow cytometry. Therefore, we selected a 6-h 100 μM H_2_O_2_ treatment to mimic I/R-induced apoptosis in vitro.

**Fig. 4:**
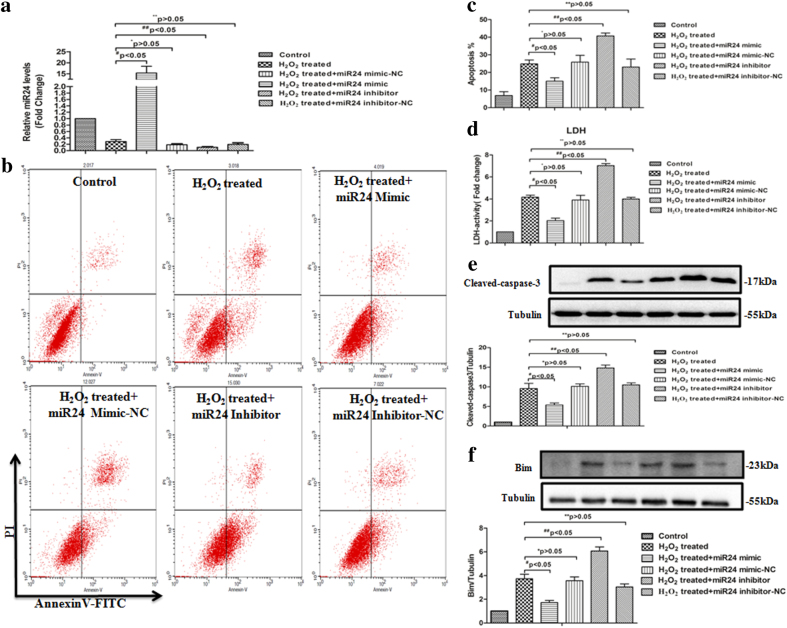
Anti-apoptotic effects of miR-24 in vitro. **a** qRT-PCR analysis of miR-24 level in H9c2 cardiomyoblasts transfected with miR-24 mimics or inhibitors or respective NC RNAs (*P > 0.05, **P>0.05, ^#^*P* < 0.05,^ ##^ *P*<0.05, *n* = 4). **b**, **c** Flow cytometric analysis of apoptosis of H_2_O_2_-exposed (100 μM, 6 h) H9c2 cells treated with miR-24 mimics or inhibitors (*P > 0.05, **P>0.05, ^#^*P* < 0.05,^##^*P*<0.05, *n* = 4). **d** Relative LDH activity in the culture media of H9c2 cells transfected with miR-24 mimics or inhibitors or respective NC RNAs (*P > 0.05,**P>0.05,^#^*P* < 0.05,^##^*P*<0.05 *n* = 5). **e**, **f** Immunoblot analysis for the expression of Bim and cleaved caspase 3 in H_2_O_2_-treated (100 μM, 6 h) H9c2 cells that had been treated with miR-24 mimics or inhibitors (*P > 0.05, **P>0.05, ^#^*P* < 0.05,^##^*P*<0.05, *n* = 3)

To demonstrate whether miR-24 can reduce apoptosis in cardiomyocytes, we performed gain- and loss-of-function experiments using miR-24 mimics/inhibitors. First, miR-24 mimics or inhibitors were transfected into H9c2 cells, and the transfection efficiency was assessed using fluorescence microscopy (Supplemental Fig. IF). Additionally, the expression level of miR-24 was detected by real-time PCR (RT-PCR), indeed, miR-24 was successfully transfected into the H9c2 cells (Fig. 4a). To detect the effects of miR-24 on H_2_O_2_-induced apoptosis in H9c2 cells, we measured the percentage of apoptotic cells in six groups of H9c2 cells using flow cytometry. As shown in Fig. 4b, c, the percentage of apoptotic cells was decreased in H9c2 cells treated with the miR-24 mimic but was increased in H9c2 cells treated with the miR-24 inhibitor. Consistent with the above observations, lactate dehydrogenase (LDH) assays showed that the miR-24 mimic decreased LDH release, whereas miR-24 inhibitors had the opposite effects (Fig. 4d). In addition, the miR-24 mimic significantly decreased the levels of cleaved caspase 3, however, the miR-24 inhibitor increased these levels (Fig. 4e) in H9c2 cells treated with 100 μM H_2_O_2_. Collectively, these findings demonstrated that miR-24 had anti-apoptotic function under conditions of oxidative stress. Since Bim has been shown to be involved in oxidative stress-induced cell death^26^, by evaluating the expression levels of Bim in H_2_O_2_-treated H9c2 cells in the presence of the miR-24 inhibitor or miR-24 mimic via western blotting, we next investigated whether Bim is a target of miR-24 in H9c2 cells. As shown in Fig. 4f, Bim was significantly upregulated in H9c2 cells treated with H_2_O_2_, and Bim levels were further increased in H9c2 cells in the presence of the miR-24 inhibitor but decreased in H9c2 cells in the presence of the miR-24 mimic.

### Anti-apoptotic effects of RIPC-EXO on H9c2 cells treated with H_2_O_2_

**Fig. 5:**
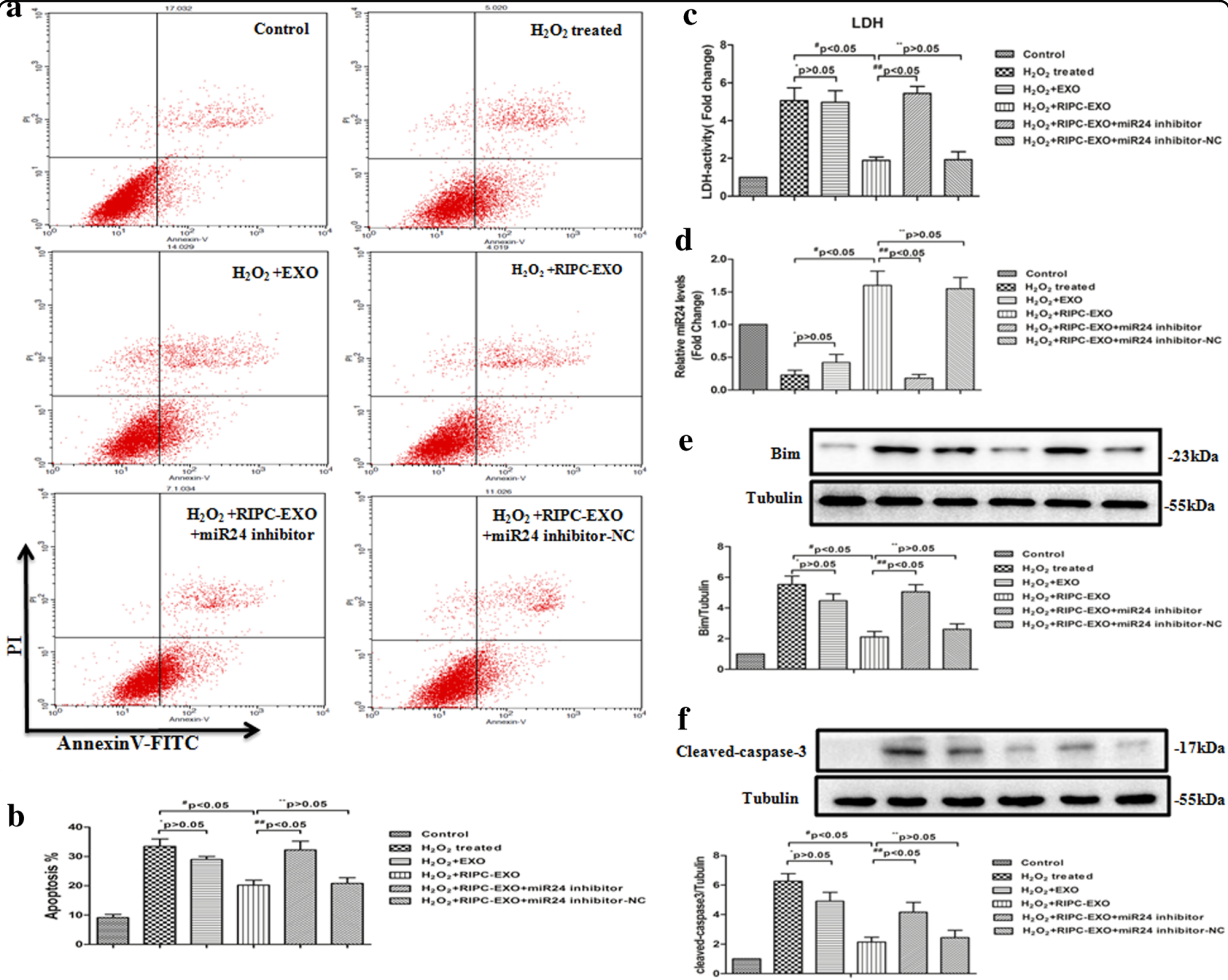
Anti-apoptotic effects of RIPC-EXO in H_2_O_2_-treated H9c2 cells. **a**, **b** Flow cytometric analysis of apoptosis in various groups of H_2_O_2_-treated H9c2 cells (*P > 0.05, **P>0.05, ^#^*P* < 0.05, ^##^*P*<0.05, *n* = 3). **c** Relative LDH activity in the culture media of H9c2 cells from the various groups. Cell damage was significantly reduced by incubation with RIPC-EXO (*P > 0.05, **P>0.05, ^#^*P* < 0.05, ^##^*P*<0.05, *n* = 3). **d** qRT-PCR analysis of miR-24 levels in H9c2 cells from the various groups (*P > 0.05,**P>0.05, ^#^*P* < 0.05, ^##^*P*<0.05, *n* = 3). **e**, **f** Immunoblot analysis of the expression of Bim and cleaved caspase 3 in various groups of H_2_O_2_-treated H9c2 cells (*P > 0.05,**P>0.05, ^#^*P* < 0.05, ^##^*P*<0.05 *n* = 3)

To determine the effects of RIPC-EXO harbouring miR-24 on H9c2 cells apoptosis induced by oxidative stress, we pretreated H9c2 cells with phosphate-buffered saline (PBS), EXO or RIPC-EXO, and subjected them to acute H_2_O_2_ or vehicle treatment, followed by the determination of apoptotic rates using flow cytometry. As shown in Fig. 5a, b, both RIPC-EXO and EXO decreased the apoptosis rate of H_2_O_2_-treated H9c2 cells, but RIPC-EXO exhibited a more pronounced effect. In line with the above findings, RIPC-EXO were more efficient in reducing necrosis of H_2_O_2_-treated cells than was EXO (Fig. 5c). Similar results were obtained regarding the levels of Bim and cleaved caspase 3 in RIPC-EXO and EXO groups (Fig. 5e, f). However, the addition of the miR-24 inhibitor counteracted the effect of RIPC-EXO-induced reduction in apoptosis. Additionally, miR-24 was significantly downregulated in H9c2 cells treated with H_2_O_2_ (Fig. 5d) and was upregulated after pre-incubation with RIPC-EXO. The RIPC-EXO-treated H9c2 cells had an approximately eightfold higher expression of miR-24 than did the EXO-treated cells (Fig. 5d). In agreement with the above findings, transferase dUTP nick end-labelling (TUNEL) assays revealed that both RIPC-EXO and EXO reduced H_2_O_2_-induced H9c2 cell apoptosis but that RIPC-EXO were more efficient. Correspondingly, the addition of the miR-24 inhibitor counteracted the apoptosis-reducing effect of RIPC-EXO (Supplemental Fig. IC-D).

### Anti-apoptotic effects of RIPC-EXO on the I/R model in vivo

Next, we explored the anti-apoptotic effects of RIPC-EXO on the rat I/R model in vivo. Rats were subjected to left anterior descending (LAD) artery occlusion for 45 min followed by reperfusion for 24 h. Unfortunately, six rats died during the experiment—one died due to an anaesthesia accident, two died of pneumothorax and three died within 3 h after surgery. We intramyocardially injected PBS, RIPC-EXO or EXO (10 µl (≈5.8 × 10^12^ particles) each) immediately after the induction of ischaemia via LAD occlusion. The expression level of miR-24 was analysed in the border zone and distant zone of hearts 24 h after I/R induction or sham surgery. The expression levels of miR-24 in the border zone of the I/R group was lower than that in the sham group, but there was no significant statistical difference between the two groups in the distant zone. However, the levels of miR-24 in the RIPC-EXO + I/R group were higher than those in the I/R group in the border zone (Supplemental Fig. IIA-B). Furthermore, RIPC improved cardiac function and reduced infarct size in the rats subjected to I/R using cardiac ultrasound and triphenyl tetrazolium chloride (TTC) staining (Supplemental Fig. IIC-F).

**Fig. 6:**
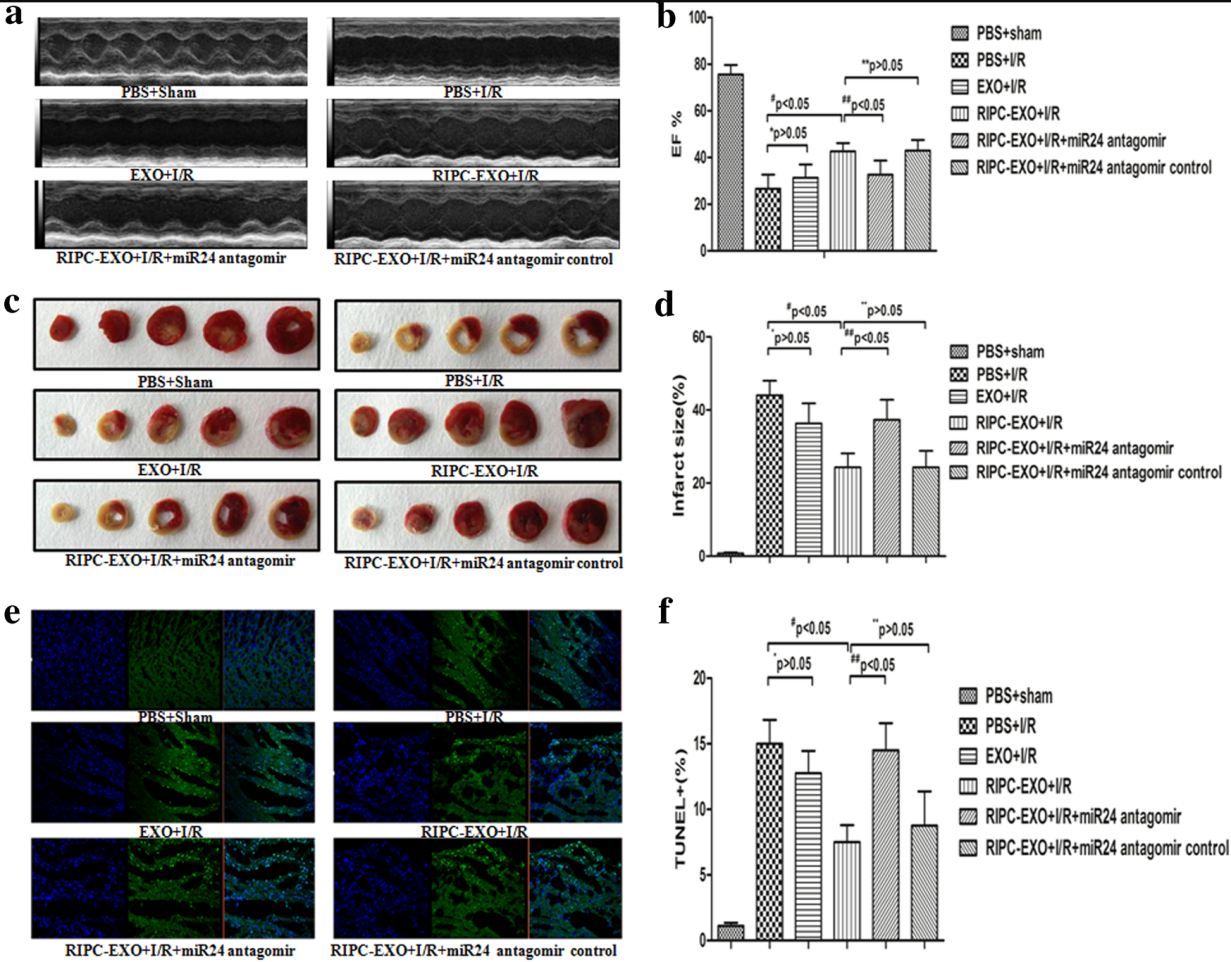
Anti-apoptotic effects of RIPC-EXO in the rat I/R model in vivo. **a** M-mode echocardiography of representative hearts 24 h after I/R. **b** LVEF was measured by echocardiography 1 day after I/R (*P > 0.05, **P>0.05, ^#^*P* < 0.05, ^##^*P*<0.05 *n* = 12). **c** Representative images of TTC staining in five sequential slices of LV from representative hearts from the various groups of rats. **d** Blinded quantification of the infarct sizes as described in the Materials and Methods section (*P > 0.05, **P>0.05, ^#^*P* < 0.05,^##^*P*<0.05 *n* = 6). **e**, **f** TUNEL assay in cardiomyocytes from the various groups. Quantification is shown in **f** (*P > 0.05, **P>0.05, ^#^*P* < 0.05, ^##^*P*<0.05, *n* = 6). LV left ventricle, EF ejection fraction

To determine whether treatment with RIPC-EXO affects rat heart functions in vivo, we assessed the rats cardiac functions before and 1 day after RIPC-EXO treatment. There was no significant changes in the left ventricular ejection fraction (LVEF) (Supplemental Fig. IE) in the rats before I/R or RIPC. However, compared to EXO administration, the administration of RIPC-EXO significantly ameliorated cardiac dysfunctions in I/R rats (Fig. 6a, b). In addition, the infarct sizes were significantly reduced from 47.3 ± 5% in the PBS + I/R group to 25.3 ± 3.1% (^#^*P* < 0.05) in the RIPC-EXO + I/R group. However, the difference in the infarct size between the PBS + I/R group and the EXO + I/R group was not significant (Fig. 6c, d). Next, using TUNEL assays, we determined whether the administration of RIPC-EXO in vivo offers protection against cardiomyocyte apoptosis induced by acute I/R. As shown in Fig. 6e, f, compared with the delivery of PBS, the delivery of EXO or RIPC-EXO to the ischaemic myocardium reduced cardiomyocyte apoptosis. However, the RIPC-EXO treatment was more effective than the EXO treatment (^#^*P* < 0.05), which suggested that RIPC-EXO exert enhanced cardioprotective effects in the context of I/R. As expected, the addition of the miR-24 antagomir counteracted RIPC-EXO-induced reduction in apoptosis.

## Discussion

RIPC elicits strong protective effects against cardiac IRI^27,28^. However, the underlying mechanisms of these effects are not precisely known. A recent study has suggested that EVs are responsible for the transmission of RIPC signals that elicit cardioprotection^2^. Our results confirmed that exosomes potentially played a central role in the cardioprotective effects of RIPC. Our results also showed that the expression of miR-24 in the exosomes from rat plasma was significantly increased after RIPC and that miR-24 in RIPC-EXO potentially played an important role in cardioprotection by attenuating cardiomyocyte apoptosis through the downregulation of Bim expression.

Recent studies have shown that exosomes derived from cardiomyocytes harbour a different kind of miRNAs, which could be transferred to surrounding endothelial cells to modulate their function^29^. Additionally, cardiomyocytes can release and take up exosomes both in vitro and in vivo^13,30,31^. In the present study, we labelled exosomes isolated from rat plasma with PKH26 and incubated them with H9c2 cells. Not only was the H9c2 cells able to take up the exosomes but the miRNA content of the exosomes was also able to enter the cytoplasm.

Cardiomyocytes apoptosis plays central role in the progression of many myocardial disorders, including I/R injury. Here H_2_O_2_-induced apoptosis was used to determine the ameliorative effect of EXO on apoptosis in H9c2 cells. Detected by flow cytometric analysis and protein levels of Bim and active-caspase 3, the induction of apoptosis caused by H_2_O_2_ could be attenuated by RIPC-EXO in H9c2 cells. Consistent with the results in vitro, the ameliorative effect of RIPC-EXO on cardiomyocytes apoptosis also is proved in myocardial IRI in vivo detected by TUNEL staining. Our results could demonstrate the beneficial effect of RIPC-EXO on myocardial apoptosis.

To elucidate the mechanism underpinning the protective role of exosomes, we extracted miRNAs from exosomes collected after RIPC and examined the expression of several miRNAs that have previously been shown to be involved in the negative regulation of apoptosis. Interestingly, we found that the expression level of miR-24 was to be significantly altered. Previous studies have shown that miR-24 can downregulate Bim expression in mice to reduce myocardial cell apoptosis during myocardial infarction^32^.

In our study, we found that the expression levels of miR-24 in H9c2 cells were significantly increased after pre-incubation with the exosomes isolated from rats that had been subjected to RIPC and that the mechanism by which miR-24 protected the heart from injury was related to the downregulation of Bim expression. The importance of miR-24 in cardioprotection was further supported by the finding that the addition of the miR-24 antagomir could counteract the miR-24-induced suppression of apoptosis. Therefore, RIPC-EXO may reduce apoptosis in H_2_O_2_-treated H9c2 cells by delivering miR-24 to these cells to negatively regulate Bim expression.

A recent study by Li et al.^16^ showed that cardioprotection elicited by RIPC was associated with the elevation of circulating miR-144 bound to Argonaute 2. In contrast, in our study, miR-144 expression was hardly altered in RIPC-EXO. To further support these findings, exosomes purified from RIPC-exposed rat plasma were treated with proteinase K, RNase or Triton X-100 at 37 °C for 45 min, after which the exosomes were examined by RT-PCR. The level of miR-24 in the exosomes was significantly decreased only after the addition of RNase and the cell membrane-disrupting agent, indicating that most of the miR-24 molecules were present in plasma exosomes. These results were consistent with the findings of Gallo A et al.^33^, who demonstrated that most of the miRNAs in serum and saliva are present in exosomes. The discrepancy between these findings may be attributed to different methods of separation of exosomes in the plasma and different time points of exosome extraction after RIPC.

To further validate our data in vivo, RIPC-EXO were injected into the hearts of rats subjected to I/R. As expected, rats subjected to I/R exhibited significantly lower cardiac ejection fraction than did sham-operated rats. Furthermore, the infarct size measured using TTC staining was lower in the RIPC-EXO-treated groups than that in the EXO-treated groups. However, our data were inconsistent with the findings of Vicencio et al.^3^, who showed a reduction in the relative infarct size by TTC staining in rats after the administration of RIPC-EXO, although the difference was not statistically significant. The exact reasons underlying these inconsistencies are not clear but could be attributed to the following: first, the timing of plasma collection after the establishment of the RIPC model was different between the two studies. Vicencio et al. collected plasma 15 min after RIPC, whereas we collected plasma from the rats immediately after RIPC. Second, the amount of plasma collected after the establishment of the RIPC model was different. Vicencio et al. collected exosomes from the plasma of one RIPC-treated rat and then injected these exosomes into another experimental rat. Considering that sufficient plasma cannot be collected from the total volume of blood from a single rat, in our study, exosomes were isolated from the plasma of two rats after RIPC and were injected into one rat. In addition, in our study, exosomes were introduced into rats by direct myocardial injection rather than by tail vein injection.

In conclusion, exosomes induced by RIPC reduced myocardial cell apoptosis resulting from I/R injury by transporting miR-24 into the myocardial cells, in which miR-24 downregulated Bim expression (Fig. 8).

### Study limitations

First, the exosomes purified using precipitation methods are far from pure, and there is no perfect way to completely purify exosomes at present, an amount of proteins and other EVs may still exist in the isolated particles. Therefore, it cannot be completely determined that the effect of heart protection is only derived from exosomes and not other EVs and proteins. Second, because there is no specific drug that can inhibit exosome secretion in vivo, it was not possible for us to demonstrate whether exosomes were involved in the protective mechanism of RIPC by directly inhibiting the release of exosomes in vivo. Third, we cannot rule out the possibility that other miRNAs or proteins in exosomes were involved in mediating the cardioprotective effect of RIPC. Fourth, the infarct area was calculated as % area of left ventricle (LV), without taking account of the area at risk. The area at risk may vary between animals, although the rats were randomised to treatment group and operator was blinded to treatment. Fifth, we did not investigate the long-term effects of exosomes on rats’ I/R injury. The increased number of exosomes may have originated from RIPC-treated hindlimbs, however, the accurate origin and reason for this increase in exosome numbers remain unknown. Sixth, the physiological characteristics of H9c2 cells are different from those of adult cardiomyocytes to some extent. Thus, our results might not accurately reflect the effects of EXO on adult cardiomyocytes. Finally, although miR-24 clearly mediated apoptosis of H9c2 cells in response to H_2_O_2_ stimulation in vitro, we only used the TUNEL assay to detect cell death in vivo. Given that the TUNEL assay does not enable the unequivocal distinction between apoptosis and necrosis, future studies should employ more apoptosis-specific methods for the in vivo detection of apoptosis in animal models of I/R.

## Conclusion

In conclusion, exosomes derived from the plasma of rats subjected to RIPC could be involved in cardiac protection against IRI via miR-24, which reduces myocardial cell apoptosis by downregulating Bim expression.

## Materials and methods

Male Sprague Dawley (SD) rats were used in this study. The protocol was approved by the Animal Care and Use Committee of the Second Affiliated Hospital of Nanchang University, China and conformed to the Guide for the Care and Use of Laboratory Animals published by the National Institutes of Health (NIH publication no. 85-23, revised 1996).

### Establishment of the RIPC model

RIPC was induced by four cycles of 5 min of limb ischaemia in rats (using a tourniquet) followed by a 5-min reperfusion as previously described^3,34^. Sham-operated rats were received as controls.

### Rat plasma preparation and exosome isolation

Blood samples were collected in K_2_-EDTA tubes (Becton Dickinson) immediately after euthanasia and processed for plasma preparation within 5 min. The samples were first centrifuged at 1500 ×* g* at 4 °C for 15 min. And the supernatants were carefully collected without disturbing the lower layers that contained platelets, and then transferred to nuclease-free tubes. Rat blood samples were collected at pre- (baseline) and post RIPC^35^. Exosomes from sham-operated animals served as control exosomes. The exosomes were isolated from plasma using ExoQuick precipitation solution^36^ (SBI, USA) according to the manufacturer’s instructions. Briefly, plasma was filtered by using a 0.22-μm pore filter and treated with thrombin to pre-clear fibrin and prevent its co-precipitation using Thrombin plasma prep for exosome precipitation (500 µl at 500 U/ml) as previously described^37^. Cell-free plasma samples were mixed with ExoQuick precipitation solution. After the mixtures were incubated for 30 min at 4 °C and centrifuged at 1500 × *g* for 30 min at 4 °C, the obtained pellets were washed with PBS, after which the exosome pellets were suspended in PBS and stored at −80 °C until use.

### Exosome identification and measurement of exosome numbers

TEM was used to investigate the morphology of exosomes. Briefly, re-suspended exosome pellet (3 μl) was set on Formvar carbon-coated 200-mesh copper TEM grids, and was subjected to uranyl acetate staining. The grids were washed with PBS and semi-dried at room temperature before observation with a TEM instrument (JSM-6701F, Tokyo, Japan), as previously described methods^38^. The diameters of the exosomes were quantified by TEM. The particle size and concentration of exosomes were analysed using NTA. Briefly, the exosomes were diluted in 1 ml PBS after isolation. PBS was used as control. And the detection thresholds were the same for all samples examined. Each sample was performed for four recordings. Additionally, the supernatants of exosome lysate were prepared, and the protein content of the exosome samples was assessed using a BCA Protein Assay Kit (Sigma, USA). Proteins extracts were separated by a 10% SDS-polyacrylamide gel electroporesis (SDS-PAGE) gel at 110 V for 60 min and transferred to a polyvinylidene difluoride (PVDF) membrane (Millipore Billerica, USA) for 80 min at 100 mA. Finally, the membrane was blocked with 5% skim milk in tris-buffered saline tween-20 (TBST) for 2 h and incubated with primary antibodies against CD63 (1:1000, SBI), CD9 (1:1000, SBI) or CD81 (1:1000, SBI) overnight, washed with TBST and further incubated with horseradish peroxidase (HRP)-linked secondary antibodies (1:5000, SBI).

### Isolation and quantification of miRNAs

Total RNA was extracted from exosomes using the miRCURY™ RNA Isolation Kit (Exiqon) following the manufacturer’s instructions, and cellular RNA was extracted using the miRNeasy Mini Kit (Qiagen). The purity of the isolated RNA was determined by the optical density 260/280 ratio using the NanoDrop ND-2000 (Thermo Scientific), and the integrity of RNA was assessed using agarose gel electrophoresis. The isolated RNA was reverse transcribed using the PrimeScript RT Reagent Kit (TaKaRa, Japan). RT-PCR was performed using the SYBR^®^ PremixEx Taq II Kit (TaKaRa) on a 7500 Fast Real-Time PCR system from Applied Biosystems (Bio-Rad, USA). Fold induction was calculated using the Ct method as follows: ΔΔCt = (Ct target miRNA − Ct celmiRNA39), and the final values were determined using 2^−ΔΔCt^.

### Exosomal degradation analysis

The exosomes purified from rat plasma were treated with proteinase K, RNase or Triton X-100 at 37 °C for 45 min. The expression levels of miRNAs were determined by qPCR.

### PKH26-labelled exosome transfer

Exosomes isolated from rat plasma after RIPC were labelled using a PKH26 red fluorescent labelling kit (Sigma, USA) according to the manufacturer’s protocols. Briefly, the exosomes were incubated with the PKH26 dye for 4 min, and the reaction was terminated by adding exosome-depleted fetal bovine serum (FBS). The exosomes were washed three times and removed excess PKH26 dye by using 100-kDa Amicon Ultra-4 (Millipore), then incubated with H9c2 cells. The NC group was the H9c2 cells, incubated only with PKH26. The incorporation of exosomes from rat plasma after RIPC into H9c2 cells was visualised by confocal microscopy. The rate of uptake of the exosomes into cells was measured by flow cytometry after various incubation times.

### H9c2 cell transfection

The miR-24 mimic, inhibitor and NC RNAs (RiboBio, Guangzhou, China) were transfected into cells with Lipofectamine 3000 (Life Technologies) according to the manufacturer’s instructions. The miR-24 mimic and mimic-NC were used at a concentration of 50 nM, whereas the miR-24 inhibitor and inhibitor-NC were used at 100 nM. Six hours after transfection, the medium was replaced. The cells were harvested for protein extraction and total RNA after 48 h. The efficiency of the mimic and inhibitor was confirmed using RT-PCR. Subsequently, cleaved caspase 3 and Bim protein expression levels were determined using western blotting.

### Apoptosis assay

**Fig. 7:**
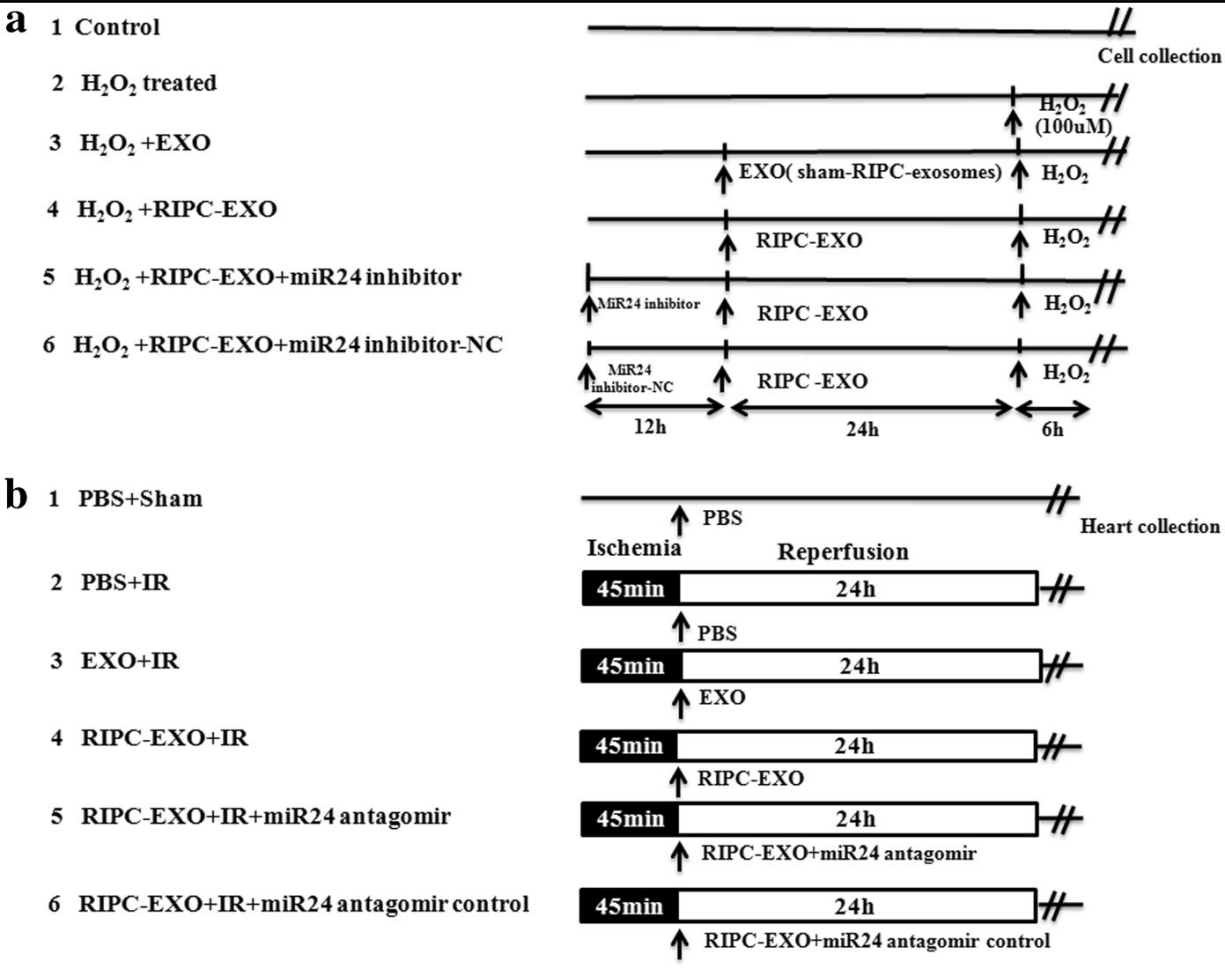
Flowchart of study design. **a** Study design for evaluating the effects of RIPC-EXO on H9c2 cells treated with H_2_O_2_. **b** Study design for systemic delivery of RIPC-EXO in the rat I/R model

H9c2 cells were purchased from American Type Culture Collection and cultured in Dulbecco’s modified Eagle medium (HyClone, USA) supplemented with 10% exosome-depleted FBS. The H9c2 cells were assigned to the following six groups: group 1 (control); group 2 (H_2_O_2_-treated); group 3 (H_2_O_2_ + EXO); group 4 (H_2_O_2_ + RIPC-EXO); group 5 (H_2_O_2_ + RIPC-EXO + miR-24 inhibitor); and group 6 (H_2_O_2_ + RIPC-EXO + miR-24 inhibit-NC). The study design is shown in Fig. 7a. Following treatment, the rate of apoptosis was determined using flow cytometry with an Annexin V/propidium iodide kit (BD Bioscience, USA) according to the manufacturer’s instructions. Additionally, western blotting was performed to evaluate the expression of apoptosis-related factors, including cleaved caspase 3 and Bim. Briefly, cell lysates were prepared by the addition of cell lysis buffer (Thermo, USA). The concentrations of cells protein were quantified by the BCA Protein Assay Kit (Sigma). Proteins extracts were separated by a 10% SDS-PAGE gel at 110 V for 60 min and transferred to a PVDF membrane (Millipore) for 80 min at 100 mA. And the membrane was blocked for 2 h with 5% skim milk in TBST, then incubated with primary antibodies against cleaved caspase 3 (1:1000, CST, USA) and Bim (1:1000, Abcam, UK) overnight, then incubated with HRP-linked secondary antibodies (1:5000, CST) for 1 h. The expression of relative protein was normalised to tubulin levels.

### In vitro LDH assay

H9c2 cells were exposed to 100 µM H_2_O_2_ for 6 h in the presence or absence of RIPC-EXO or H9c2 cells transfected with the miR-24 inhibitor or mimic. LDH release was used as a marker for cell injury, and quantified using CytoTox-ONE Homogeneous Membrane Integrity Assay (Promega) according to the manufacturer’s instructions.

### Generation of the rat myocardial I/R model

Rats were subjected to myocardial I/R as previously described^39^. Briefly, SD rats weighing 200–250 g were mechanically ventilated with adequate anaesthesia with pentobarbital (50 mg/kg, intraperitoneally). The chest of rat was opened through lateral thoracotomy, then the heart was exposed via pericardiotomy. A 6-0 nylon suture was placed under the LAD and then ligated a snare for reversible LAD occlusion through a small plastic (PE10) tube. The LAD was occluded for 45 min, and then reperfusion was initiated by gently removing the plastic tube. Finally, the chest was closed, and the rat was allowed to recover. The rats were sacrificed 24 h after the reperfusion for histological assessment. The rats were randomised to treatment group and the operator was blinded to treatment.

### Intramyocardial delivery of exosomes

Delivery of exosomes to the myocardium was performed immediately after the induction of ischaemia in rats by LAD ligation. The exosomes were collected from plasma of rats in RIPC-EXO group and EXO group, respectively, and then calculated the exosome concentration of each group by Nanosight, and the other group of rats were injected with the same number and volume of exosomes (≈5.8 × 10^12^ particles diluted in 10 μl PBS) as the EXO group.The rats were assigned to six groups based on the injection as follows: group 1 (PBS + sham), intramyocardial delivery of 10 µl PBS to sham rat; group 2 (PBS + I/R), intramyocardial delivery of 10 µl PBS to I/R rat; group 3 (EXO + I/R), intramyocardial delivery of 10 µl exosomes purified from the plasma from sham-operated rats; group 4 (RIPC-EXO + I/R), intramyocardial delivery of 10 µl exosomes purified from the plasma of RIPC-treated rats; group 5 (RIPC-EXO + I/R + miR-24 antagomir), intramyocardial delivery of 10 µl exosomes purified from the plasma of RIPC-treated rats and 10 nmol of the miR-24 antagomir; and group 6 (RIPC-EXO + I/R + miR-24 antagomir control). Figure 7b shows the study design.

### Echocardiography

**Fig. 8:**
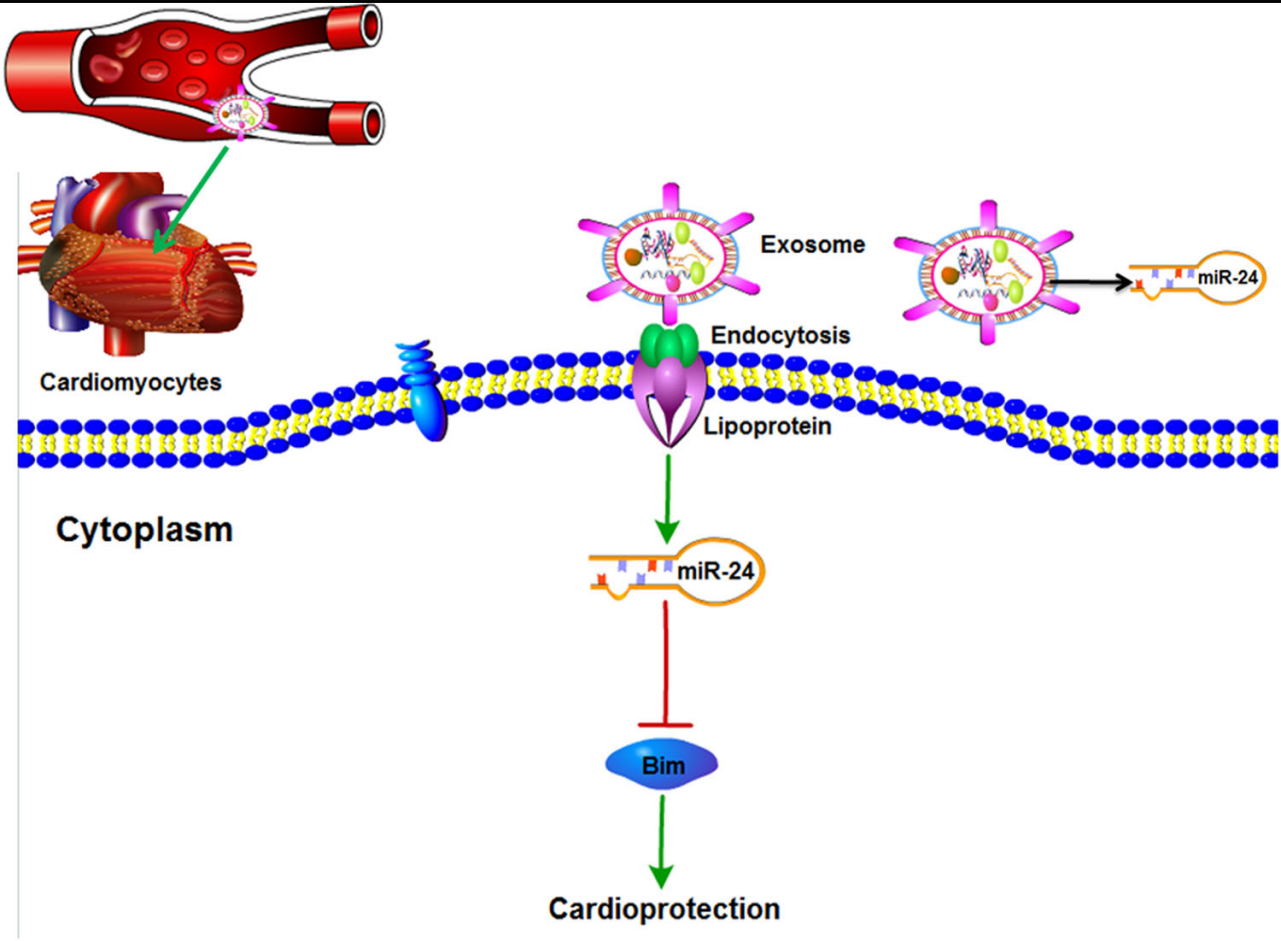
Schematic representation of a working model. Under oxidative stress, RIPC-treated rats secrete a considerable number of exosomes, which could be taken up by cardiomyocytes, and miR-24 contained in RIPC-EXO inhibits cardiomyocyte apoptosis by downregulating the expression of Bim, thereby resulting in cardiac protection

Echocardiography was performed to evaluate cardiac function before operation and 1 day after I/R. The operator was blinded to treatment. Transthoracic echocardiography was performed using a Vevo 2100 Imaging System (Visual Sonics, USA) according to previously described methods^40,41^. Briefly, rats were anaesthetized with pentobarbital. At the level of the papillary muscles, a two-dimensional short-axis view of the LV was obtained. The LVEF was calculated by measuring LV end-systolic volume and LV end-diastolic volume using a modified Simpson’s method (Fig. 8).

### Histology

To detect the number of apoptotic cardiomyocytes, rat hearts were removed 1 day after I/R. Then hearts were fixed with 30% sucrose, then embedded in optimal cutting temperature compound (OCT) and sectioned after frozen. The TUNEL assay was performed by using the DeadEnd™ Fluorometric TUNEL System (Promega) according to the manufacturer’s protocol. The slides were mounted with 4′,6-diamidino-2-phenylindole. Staining was analysed using a Zeiss 710 Laser Scanning Microscope (Carl Zeiss, Thornwood, NY). The apoptotic cardiomyocytes were quantified and classified according to their TUNEL-positivity in a given field (×200).

### Measurement of infarct size

One day after coronary ligation, the rats were anaesthetised. LVs were isolated and cut into five ~1 mm pieces, in which the first cut was at the ligation level. The LV slices were stained with 1.5% TTC at 37 °C for 30 min, and fixed in 4% formalin at 4 °C overnight^42^. The infarct area was demonstrated as a white area, while the viable myocardium was stained red. The photographs were captured on both sides of each section. Infarct sizes were evaluated in a blinded manner using ImageJ software.

### Statistical analysis

The data are presented as the mean ± standard error of the mean. For all comparisons, statistical significance was determined using one-way analysis of variance, followed by post hoc tests (Newman–Keuls) where appropriate. A *P*-value <0.05 was considered statistically significant.

## Electronic supplementary material

Supplement.docx

Fig-S-I.jpg

Fig-S-II.jpg
