# Supplementary material for: Plasma exosomes induced by remote ischaemic preconditioning attenuate myocardial ischaemia/reperfusion injury by transferring miR-24
Source: Cell Death Dis. 2018 Feb 23;9(3):320. doi: 10.1038/s41419-018-0274-x (PMC5833738; doi:10.1038/s41419-018-0274-x)
Supplement: Supplementary file 1 — Supplement.docx [file 41419_2018_274_MOESM1_ESM.docx]

Supplemental Figure I. (A) Apoptosis and necrosis were analysed by flow cytometry using the Annexin V/PI assay in H9c2 cells treated with H_2_O_2_ at the indicated concentrations for 4 h. Necrosis, PI+; apoptosis, Annexin V+/PI-. Data are shown as the means ± SDs (n = 3) (^#^P < 0.05 vs control). (B) H9c2 cells were treated with H_2_O_2_ at 100 μM for the indicated times to induce apoptosis. Apoptotic and necrotic cells were analysed by flow cytometry using the Annexin V/PI assay. (C-D) Apoptosis was detected using the TUNEL assay in H9c2 cells treated as described in Fig. 6. Blue, DAPI-stained nuclei; green, TUNEL-positive nuclei. Data are shown as the means ± SDs (n = 4). (E) EFs were measured using echocardiography 1 day before I/R. Data were collected 1 day before I/R in a blinded fashion. There were no significant differences in EFs between the groups (^*^P > 0.05). (F) miR-24 mimic transfection efficiency was determined using fluorescence microscopy.

Supplemental Figure II. (A-B) miR-24 levels were analysed using qPCR on RNA extracted from the border zone and distant zone of hearts 24 h after I/R or sham surgery (*P > 0.05, ^#^P < 0.05, n = 3). (C) Representative images of hearts by M-mode echocardiography 24 h after I/R. (D) EF in the different groups was measured using echocardiography. Data were collected 24 h after I/R in a blinded fashion (*P > 0.05, ^#^P < 0.05, n = 6). (E) Representative images of TTC staining in five continuous slices of LV from representative rat hearts from different groups. All staining was performed on heart tissues 24 h after I/R. (F) Blinded quantification of infarct size was performed as described in the Materials and methods section (*P > 0.05, ^#^P < 0.05, n = 4)
