# Supplementary figures and images for: Plasma exosomes induced by remote ischaemic preconditioning attenuate myocardial ischaemia/reperfusion injury by transferring miR-24
Source: Cell Death Dis. 2018 Feb 23;9(3):320. doi: 10.1038/s41419-018-0274-x (PMC5833738; doi:10.1038/s41419-018-0274-x)

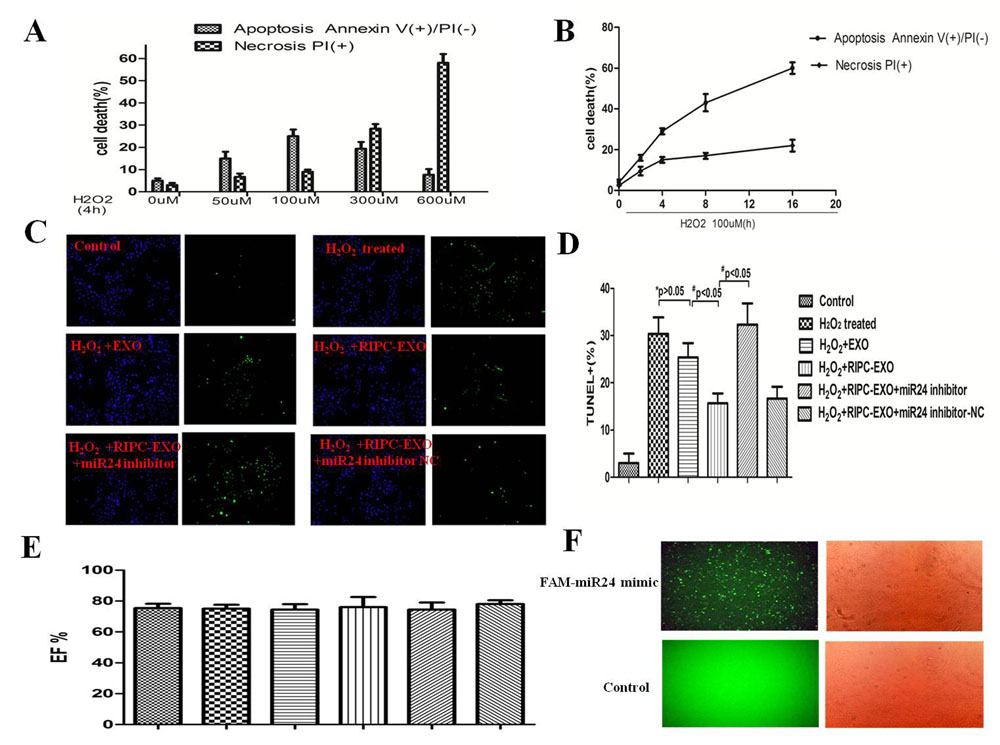

Supplement: Supplementary file 2 — Fig-S-I.jpg [file 41419_2018_274_MOESM2_ESM.jpg]

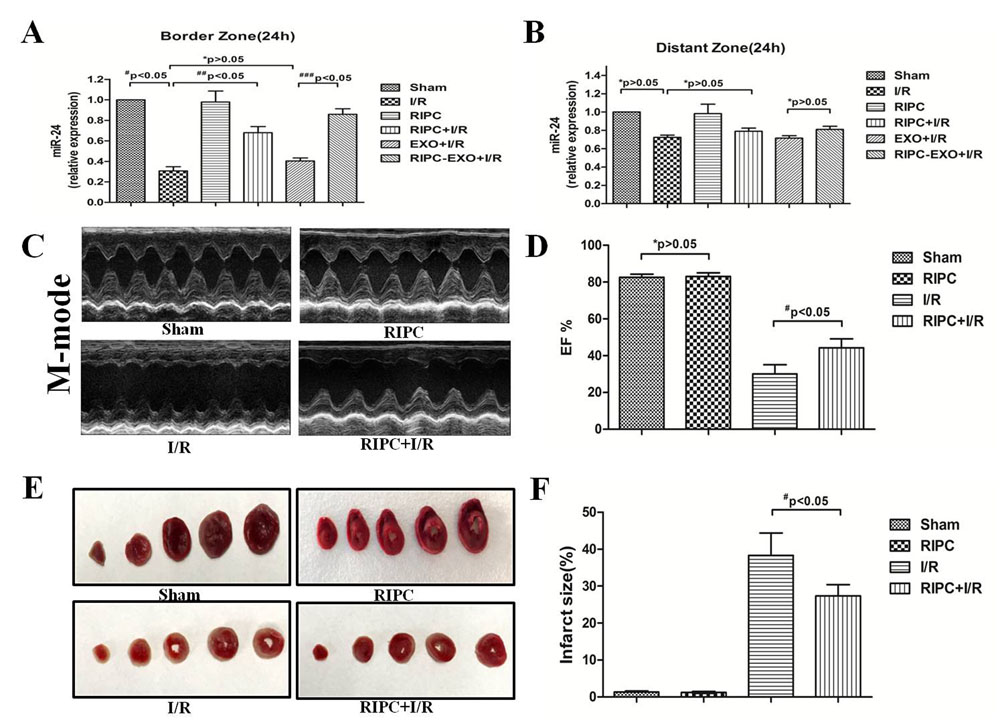

Supplement: Supplementary file 3 — Fig-S-II.jpg [file 41419_2018_274_MOESM3_ESM.jpg]
